# Editorial: The bidirectional relationship between sleep and neuroendocrinology

**DOI:** 10.3389/fendo.2024.1372967

**Published:** 2024-01-26

**Authors:** Deborah Suchecki, Peter Meerlo, T. John Wu

**Affiliations:** ^1^ Department of Psychobiology, Escola Paulista de Medicina, Universidade Federal de São Paulo, São Paulo, Brazil; ^2^ Groningen Institute for Evolutionary Life Sciences, University of Groningen, Groningen, Netherlands; ^3^ Department of Gynecologic Surgery and Obstetrics, Uniformed Services University of the Health Sciences, Bethesda, MD, United States

**Keywords:** sleep, orexin, social jetlag, metabolic syndrome, inflammation, estrogen, insomnia, sleep apnea

Sleep is a behavior implicated in numerous functions, from immunological competence to learning and memory (1–3). The activity of many neuroendocrine systems is dependent on the sleep-wake rhythm; sleep stimulates the release of anabolic hormones (1, 4, 5) and inhibits the secretion of catabolic ones, such as glucocorticoids (1, 6), whereas waking has the opposite effects. Conversely, peripheral hormones and neuropeptides modulate sleep under both basal and stressful conditions (7–9), indicating a complex bidirectional relationship between endocrine and sleep-wake regulations. Chronic impairment of sleep or reduction of sleep time may both be a cause and a consequence of disturbed endocrine activity, and together they can have profound effects on brain functions, cognition, and emotional regulation, both in humans (10, 11) and rodents (12–15).

This Research Topic of Frontiers in Endocrinology – Neuroendocrine Session – presents six original papers investigating the relationship between sleep and (neuro)endocrine influence on metabolic, immunological and cognitive functions. The study by Ma et al. explores the role of orexins in fear conditioning-induced sleep impairment employing anatomical, functional and pharmacologic techniques. They demonstrated that mice exposed to fear conditioning displayed impairment of both REM and NREM sleep and greater activation of orexin pathway to the ventrolateral preoptic area (VLPO). Activation of this orexinergic pathway by optogenetic manipulation, as well as infusion of orexin-A into the VLPO, led to similar sleep impairments.

Due to a combination of biological (late chronotype), social (e.g., early school start time) and technological exposure (e.g., internet, social media) factors, adolescents tend to compensate for the weekdays’ sleep debt on weekends. The difference in sleep time between weekdays and weekends is defined as social jet leg (SJL) (16, 17). In an epidemiological study, SJL was shown to lead to increased body mass index, in individuals aged between 16 and 65 years (18). In their study on Brazilian adolescents of both sexes, aged 9 to 15 years, Pompeia et al. investigated the impact of SJL on these adolescents’ cardiometabolic health. They found that girls are especially vulnerable to the effects of SJL on altering cardiovascular and metabolic markers. In a cohort of 352 adolescents of both sexes, 16- to 19-year-olds, plasma and salivary inflammatory cytokines and metabolic-related hormones were related to sleep parameters, such as bedtime (before or after midnight), sleep duration and SJL. Greater sleep debt and SJL had a major impacts on these parameters, increasing the levels of pro-inflammatory markers, as well as adiponectin and leptin (Alqaderi et al.).

Pre-clinical and clinical studies indicate that sleep disturbances and poor sleep quality (19) increase plasma levels of corticosterone (20–23) or cortisol (24, 25). Sexual dimorphism in cortisol levels is a well-known biological phenomenon, with women displaying higher cortisol levels throughout the day (26) and in response to challenges (27, 28). Likewise, insomnia shows sex differences and greater incidence is reported in women than men (29). Mazgelyté et al. reported a positive correlation between poorer subjective sleep quality and higher hair cortisol levels in a group of peri- and postmenopausal women, suggesting that poor sleep quality is connected to chronically elevated cortisol levels. Moreover, dysregulation of sex hormones, as seen in peri- and postmenopausal women, seems to be related to poor sleep quality and the overall stress load as shown in the accumulation of cortisol in hair over a period of time. Indeed, middle aged women who underwent oophorectomy displayed worse objective and subjective sleep than age-matched ovary-intact women. Hormone replacement therapy mitigated the sleep effects in the women who underwent oopphorectomy. In addition, increased sleep latency and reduced sleep efficiency were implicated in impaired declarative memory and reduced volume of the anterolateral entorhinal cortex.

The last paper in this volume sought to set apart the role of nocturnal hypoxemia on glycosylated hemoglobin in sleep apnea patients. Obstructive sleep apnea (OSA) is a sleep breathing disorder leading to metabolic syndrome (30). In Mahmoud et al. study, glycosylated hemoglobin was associated with reduced time of oxygen saturation, but not with apnea-hypopnea index, in OSA patients. This result suggests that nocturnal hypoxemia might be a risk factor for the development or worsening of diabetes in OSA patients.

Together, the studies included in this Research Topic demonstrate that poor or inadequate sleep impacts metabolic, immunological and cognitive functions, irrespective of age and sex, although females appear to be at greater risk of sleep-induced symptoms of metabolic syndrome.

## Author contributions

DS: Writing – original draft, Writing – review & editing. PM: Writing – original draft, Writing – review & editing. TW: Writing – original draft, Writing – review & editing.
